# Report of the Proceedings of the Military Tract Medical Association, Held at Aledo, Mercer County, Ill., Dec. 13th, 1869

**Published:** 1870-02

**Authors:** J. R. Webster, V. C. Secord

**Affiliations:** President; Secretary pro tem.


### REPORT OF THE PROCEEDINGS OF THE MILITARY TRACT MEDICAL ASSOCIATION, HELD AT ALEDO, MERCER COUNTY, ILL., DEC. 13TH, 1869.

Pursuant to adjournment, the ninth semi-annual meeting of the Military Tract Medical Association met at Aledo, Mercer County, Ill., December 13th, 1869.

The meeting was called to order by the President, Dr. J. R. Webster.

The Secretary not being present, Dr. V. C. Secord was elected Secretary *pro tem.*

The following members were present:—Drs. J. R. Webster, of Monmouth; H. S. Hurd, Galesburg; S. P. Breed, Princeton; H. Nance, Kewanee; J. H. Brewer, Monmouth; W. D. Craig, Aledo; Wm. Byms, Windsor; J. C. Smiley, Kewanee; and V. C. Secord, Galva.

The reading of the minutes of the last meeting were read and approved.

The following gentlemen were nominated and elected members of this Association:—Drs. H. C. Graham, of Windsor, Mercer County; J. V. Frazer, Viola, Mercer County; Thomas Willots, New Boston; J. C. Kilgore, Little York; J. W. Clowes, Windsor; E. L. Marshall, Keithsburg; T. H. Brass, New Boston; J. P. Boyd, Millersburg; E. L. McKennie, Viola, Mercer County; J. K. L. Duncan, Hanlet; S. G. Hodges, Sunbeam; W. F. Suiter, New Boston; G. G. Irwin, Aledo, Mercer County; J. F. Wood, Aledo; and J. F. McCutchen, Norwood.

Dr. Brewer, of Monmouth, read a very able and lengthy essay on therapeutics and pharmacy. A vote of thanks was given the Dr., and instructions to the Secretary to have a copy of the essay sent to the CHICAGO MEDICAL EXAMINER, for publication.

Dr. S. P. Breed, of Princeton, Chairman of Committee on Surgery, read a very interesting paper on dislocation of elbow-joint, complicated with fracture of the internal condyle of humerus extending into the joint; also a case of longitudinal fracture of the humerus. He gave a very interesting and minute description of a case of oseto-sarcomatous tumor, which he removed from the posterior part of the leg, also the removal of a tumor from the nates, and the extraction of nævus from the forehead of a child. He also exhibited a specimen of calculus, weighing 27 drachms, at a *post mortem* examination, and a *polypus* from the heart of the same patient.

The Dr. dwelt at length on the ganglionie cell formation of the nerves, which was very interesting and instructive, and was listened to with great attention and interest by the members of the Association, especially our worthy President, Dr. Webster.

A vote of thanks was given to Dr. Breed for his valuable contribution, and that the Dr. be permitted to retain his essay for further investigation and research, and report said investigations at some future meeting.

Dr. Hiram Nance, of Kewanee, addressed the Association at some length, giving his treatment of several diseases, *viz.:* dysentery of children, puerperal fever, syphilis, and purpura hemorrhagica, for which a vote of thanks was given.

It was moved and seconded that the Secretary furnish a copy of the proceedings of this meeting, and send them to the CHICAGO MEDICAL EXAMINER, for publication.

The committees being duly appointed, it was, on motion of Dr. Hurd, and seconded by Dr. Breed, that we adjourn to meet in Galesburg, on the third Tuesday in May, 1870.

J. R. WEBSTER, *President*.

V. C. SECORD, *Secretary pro tem.*

